# Initial combination therapy versus step-up therapy in treatment to the target of remission in daily clinical practice in early rheumatoid arthritis patients: results from the DREAM registry

**DOI:** 10.1186/s13075-016-0962-9

**Published:** 2016-03-08

**Authors:** L. M. M. Steunebrink, G. A. Versteeg, H. E. Vonkeman, P. M. ten Klooster, H. H. Kuper, T. R. Zijlstra, P. L. C. M. van Riel, M. A. F. J. van de Laar

**Affiliations:** Arthritis Center Twente, Department of Rheumatology, Medisch Spectrum Twente, PO Box 50 000, 7500 KA Enschede, The Netherlands; Department Psychology, Health & Technology, University of Twente, Enschede, The Netherlands; Department of Rheumatology, Isala Klinieken, Zwolle, The Netherlands; Department of Rheumatology, Bernhoven, Uden, The Netherlands

**Keywords:** Treat-to-target strategies, Early rheumatoid arthritis, Remission, DAS28

## Abstract

**Background:**

Treat to target (T2T) is widely accepted as the standard of care for patients with rheumatoid arthritis (RA) and has been shown to be more effective than traditional routine care. The objective of this study was to compare the effectiveness of two T2T strategies in patients with early RA: a step-up approach starting with methotrexate (MTX) monotherapy (cohort I) versus an initial disease-modifying antirheumatic drug combination approach (cohort II).

**Methods:**

A total of 128 patients from cohort II were case–control-matched with 128 patients from cohort I on gender, age, and baseline disease activity. Twelve-month follow-up data were available for 121 patients in both cohorts. The primary outcome was the proportion of patients having reached at least one 28-joint Disease Activity Score (DAS28) score <2.6 (remission) during 12 months of follow-up. Secondary outcomes were time until remission was achieved and mean DAS28 scores at 6- and 12-month follow-up.

**Results:**

After 12 months of follow-up, remission was reached at least once in 77.3 % of the patients in cohort II versus 71.9 % in cohort I (*P* = 0.31). Median time until first remission was 17 weeks in cohort II versus 27 weeks in cohort I (*P* = 0.04). A significant time by strategy interaction was found in mean DAS28 scores. Post hoc analysis revealed a significant difference in mean DAS28 scores between both cohorts at 6 months (*P* = 0.04), but not at 12 months (*P* = 0.36).

**Conclusions:**

The initial combination strategy resulted in a comparable remission rate after 1 year but a significantly shorter time until remission. At 6 months, mean DAS28 scores were lower in patients with initial combination treatment than in those with step-up therapy. At 12 months, no significant differences remained in mean DAS28 scores or the proportion of patients in remission.

## Background

Treat to target (T2T) is widely accepted as the standard of care for patients with rheumatoid arthritis (RA) and is now recommended in national and international guidelines [1, 2]. Under the controlled conditions of randomized clinical trials, T2T aimed at remission or low disease activity leads to good clinical outcomes [3–5]. Furthermore, it has been shown that T2T is more effective than traditional routine care, especially when a protocol treatment strategy is used [6]. Recently, it was shown that T2T also leads to more rapid and higher 28-joint Disease Activity Score (DAS28) remission rates and larger improvements in functional ability and patient assessments than usual care in daily clinical practice [7]. Previous clinical trials have additionally compared the effectiveness of different disease-modifying antirheumatic drug (DMARD) strategies, including initial monotherapy, initial combination therapy, or step-up combination therapy with and without the use of corticosteroids or biologics [5, 8–13]. On the basis of these trials, initial combination therapy appears to be more effective than initial monotherapy [5, 12, 14], while the combination of methotrexate (MTX) monotherapy with high-dose step-down prednisone seems to have equally good results [11, 15]. However, the optimal treatment protocol is still not clear.

A challenge in the field of RA management remains the implementation of T2T strategies in daily clinical practice. Healthcare professionals may be reluctant to prescribe complex combination therapies, including high-dose corticosteroids, because of concerns about patient compliance and unfavorable side effects [16]. Moreover, time constraints and problems with organizational structures and processes can complicate successful implementation of T2T in daily clinical practice [17]. T2T starting with MTX monotherapy was successfully implemented in the first Dutch Rheumatoid Arthritis Monitoring (DREAM) registry remission induction cohort [18–20]. Despite very promising results, T2T did not have the same impact on all patients [21]. A relevant proportion of patients still did not reach clinical remission within 12 months of treatment [18]. DREAM registry remission induction cohort I was used in the first observational study to describe the results of implementing a T2T strategy in daily clinical practice. Subsequent studies using different T2T strategies are needed to establish which strategy gives the optimal result in daily clinical practice.

Consequently, a second inception cohort was started (cohort II), in which patients were treated with initial combination DMARD therapy consisting of high-dose MTX and hydroxychloroquine (HCQ) with an optional intramuscular triamcinolone acetonide (TCA) injection at the discretion of the rheumatologist. The aim of the present study was to compare the clinical outcomes of both treatment strategies after 1 year of follow-up.

## Methods

### Data selection and study design

In this study, we used data from the ongoing DREAM registry remission induction strategies I and II, two observational, multicenter cohorts established in 2006 and 2012, respectively. In both cohorts, all adults aged ≥18 years with a clinical diagnosis of RA and a disease duration (time from the diagnosis to the start of therapy) <1 year were enrolled consecutively immediately after a clinical diagnosis of RA. For this study, data from two hospitals were used: Medisch Spectrum Twente in Enschede and Isala Klinieken in Zwolle, both in the Netherlands. Both treatment strategies were in line with clinical practice and complied with current guidelines for treatment of RA. Exclusion criteria for both cohorts were use of prednisolone ≥10 mg/day or previous or current treatment with DMARDs. The medical ethics committees of the Medisch Spectrum Twente, Enschede and Isala, Zwolle hospitals determined, in accordance with Dutch law, that no ethical approval was required, because all data were collected in the course of regular daily clinical practice. Nonetheless, patients were completely informed about the study, and informed consent was obtained from each patient.

At the time of the present analysis, 137 patients had a follow-up of at least 1 year in cohort II. For the aim of this quasi-experimental study, a total of 128 patients from cohort II could be individually matched with 128 patients from cohort I on sex, age (±5 years) and baseline disease activity (±0.5 on the DAS28).

### Treat-to-target protocol

#### T2T protocol in cohort I

Patients were evaluated at 0, 8, 12, 20, 24, 36, and 52 weeks and every 3 months thereafter. Per-protocol treatment advice was an initial monotherapy of 15 mg/week MTX, with folic acid taken on the second day after MTX. In case of insufficient response (DAS28 ≥ 2.6) at the first subsequent time point, the following per-protocol treatment steps were advised. After 2 months, the MTX dosage was increased to 25 mg/week; after 3 months, sulfasalazine (SSZ) 2000 mg/day was added. After 20 weeks, the SSZ dosage was increased to 3000 mg/day. In accordance with Dutch reimbursement regulations, anti–tumor necrosis factor (anti-TNF)-α treatment was prescribed at week 24 for patients whose DAS28 remained ≥3.2. In these cases, SSZ was replaced with subcutaneous administration of 40 mg of adalimumab every 2 weeks. At week 36, the frequency of adalimumab was increased to 40 mg/week for patients with DAS28 ≥ 2.6. At week 52, adalimumab was exchanged for etanercept 50 mg/week for patients with DAS28 ≥ 3.2. If at any time point the target of DAS28 < 2.6 was met, medication was held constant. In case of sustained remission (≥6 months), medication was gradually reduced and eventually discontinued. In case of a disease flare (DAS28 ≥ 2.6), the last effective medication or medication dose was restarted and treatment could subsequently be intensified. In individual patients with contraindications to specific medication, deviations from the protocol were allowed. In patients with an allergy to sulfa drugs (sulfonamides), SSZ was replaced by oral HCQ at a dosage of 400 mg/day. Concomitant treatment with nonsteroidal anti-inflammatory drugs, prednisolone at a dosage of ≤10 mg/day, and intraarticular corticosteroid injections was allowed at the discretion of the attending rheumatologist. On clinical indication, the attending rheumatologist was free to diverge from the medication schedule at any time. Further details of the study protocol are reported elsewhere [18].

#### T2T protocol in cohort II

Patients were evaluated at months 0, 2, 4, and 6 and every 3 months thereafter. Treatment advice was an initial combination therapy of MTX 20 mg/week and HCQ 200 mg twice daily. As bridging therapy, an optional intramuscular triamcinolone injection to a maximum dosage of 120 mg could be given. After 1 month, the MTX dosage was increased to 25 mg/week, independent of disease activity. All patients who started MTX also received folic acid on the second day after MTX. After 2 months, in case of persistent disease activity (DAS28 ≥ 2.6), the MTX dosage was further increased to 30 mg/week and an extra optional intramuscular triamcinolone injection could be administered. After 4 months, in case of moderate to high disease activity (DAS28 ≥ 3.2), a TNF inhibitor (adalimumab, etanercept, or infliximab) was added. In case of low disease activity (2.6 < DAS28 < 3.2), the attending rheumatologist could choose between SSZ 2000–3000 mg/day or an intramuscular triamcinolone injection.

Similarly to cohort I, disease activity was assessed using the DAS28 at each subsequent visit. If remission (DAS28 < 2.6) had not been reached, treatment was intensified. If the patient was in DAS28 remission, medication was held constant. In case of sustained remission (≥6 months), medication was tapered and eventually discontinued according to a predetermined tapering schedule. In case of disease flare (DAS28 ≥ 2.6), the last effective medication or medication dosage was restarted and treatment could subsequently be intensified. On clinical indication, the attending rheumatologist was free to diverge from the medication schedule at any time. For patients with contraindications to specific medications, deviations from the protocol were also allowed.

### Assessments

At each assessment, data were collected on various clinical and patient-reported outcome measures, including measures of disease activity, health-related quality of life, physical function, and laboratory measures. Disease activity was assessed by trained rheumatology nurses using the DAS28, consisting of swollen and tender joint counts in 28 joints, the erythrocyte sedimentation rate (ESR), and a 100-mm visual analogue scale (VAS) on general health (0 = “very good” and 100 = “very bad”) [22]. The Health Assessment Questionnaire Disability Index (HAQ-DI) was used to assess physical function [23]. Furthermore, patients rated their pain on a 100-mm VAS (0 = “no pain” and 100 = “unbearable pain”) and completed the 36-item Short Form Health Survey (SF-36) for assessment of their current physical and mental health status [24].

### Outcome measures

The primary outcome was the proportion of patients having reached at least one DAS28 < 2.6 (DAS28 remission) after 1 year of follow-up. Secondary outcomes were time until remission was achieved and changes in mean DAS28 scores at 6 months and 1 year.

### Statistical analysis

All statistical calculations were performed using the statistical software package IBM SPSS version 20.0 for Windows (IBM, Armonk, NY, USA). Missing values for ESR (1.8 %) and well-being (0.3 %) were imputed on the basis of swollen and tender joint counts and C-reactive protein (CRP) using single imputation with the expectation–maximization method in SPSS. Descriptive statistics for normally distributed variables or categorical variables were reported as frequencies, means, and standard deviations. If variables were not normally distributed, the median with the corresponding interquartile range was reported. To test for any baseline differences, we performed independent *t* tests for normally distributed variables, Mann–Whitney *U* tests for nonnormally distributed variables, and *χ*^2^ tests for categorical variables. Differences in the proportion of patients achieving remission were tested using *χ*^2^ tests. To compare time until first moment of DAS28 remission, Kaplan-Meier survival analysis was used with log-rank testing for between-group differences. The course of DAS28 over time was compared between groups by specifying a linear mixed model with strategy and time as fixed factors. As the visit schedules were slightly different between both cohorts, only baseline, 6-month, and 12-month data were used for this analysis. An ante-dependence structure was used as a model for the covariance matrix because it gave the best fit to the data. Post hoc analysis of covariance (ANCOVA) was performed with baseline DAS28 score as a covariate. For all tests, *P* < 0.05 was considered to be significant.

## Results

Table 1 summarizes baseline patient characteristics of both cohorts. All patients had active disease with a mean DAS28 of 4.8 ± 1.1 in cohort II versus 4.5 ± 1.1 in cohort I. Twelve-month data were available for 121 patients (94.5 %) in both cohorts. In cohort I, seven patients were lost to follow-up, of whom one had received another diagnosis (systemic lupus erythematosus), four were lost to follow-up for unknown reasons, and two had no 12-month visit (but were still in the cohort). In cohort II, seven patients were lost to follow-up, of whom one died, one achieved medication-free remission, one was misdiagnosed as having arthritis, two were lost to follow-up for unknown reasons, and two had no 12-month visit (but were still in the cohort).

**Table 1:** Baseline characteristics of patients in both cohorts

Characteristics	Cohort I (*n* = 128)	Cohort II (*n* = 128)	*P* value
Female sex, *n* (%)	79 (61.7 %)	79 (61.7 %)	1.00
Age, years, mean ± SD	59.1 ± 13.0	59.5 ± 12.8	0.81
DAS28-ESR, mean ± SD	4.5 ± 1.1	4.8 ± 1.1	0.02
ESR, mm/h, median (IQR)	22.0 (14.0–41.0)	29.0^a^ (14.0–45.0)	0.20
CRP, mg/L, median (IQR)	10.0^b^ (5.0–22.0)	11.5 (4.3–24.8)	0.79
Anti-CCP–positive, *n* (%)	74^c^ (58.3 %)	77^c^ (60.2 %)	0.93
RF-positive, *n* (%)	62 (48.4 %)	76 (59.4 %)	0.11
SJC, *n*, median (IQR)	6.0 (3.0–9.0)	5.0 (2.0–10.0)	0.24
TJC, *n*, median (IQR)	3.0 (1.0–7.0)	4.0 (2.0–10.0)	0.03
HAQ-SDI, median (IQR)	1.19^d^ (0.88–1.63)	1.0^e^ (0.38–1.50)	<0.01
VAS well-being, median (IQR)	50.0 (28.3–65.0)	51.0 (35.0–70.0)	0.29
VAS pain, median (IQR)	50.0^f^ (39.8–64.0)	62.0^g^ (49.0–75.0)	<0.01
SF-36-PCS, mean ± SD	38.1 ± 7.6^h^	37.3 ± 9.2^i^	0.51
SF-36-MCS, mean ± SD	40.7 ± 7.4^j^	44.9 ± 11.9^k^	0.00
BMI, kg/m^2^, mean ± SD	26.5 ± 4.8^*l*^	26.0 ± 4.1^m^	0.39

*DAS28-ESR* Disease Activity Score in 28 joints using erythrocyte sedimentation rate, *CRP* C-reactive protein, *TJC* tender joint count, *SJC* swollen joint count, *HAQ-SDI* Health Assessment Questionnaire Disability Index (standard scoring), *SF-36* 36-item Short Form Health Survey (version 2), *PCS* Physical Component Summary, *MCS* Mental Component Summary, *BMI* body mass index, *RF* rheumatoid factor, *CCP* cyclic citrullinated peptide, *VAS* visual analogue scale

^a^ *n* = 127

^b^ *n* = 121

^c^ *n* = 127

^d^ *n* = 102

^e^ *n* = 87

^f^ *n* = 122

^g^ *n* = 72

^h^ *n* = 105

^i^ *n* = 86

^j^ *n* = 105

^k^ *n* = 86

^*l*^ *n* = 126

^m^ *n* = 119

### Remission

**Fig. 1:**
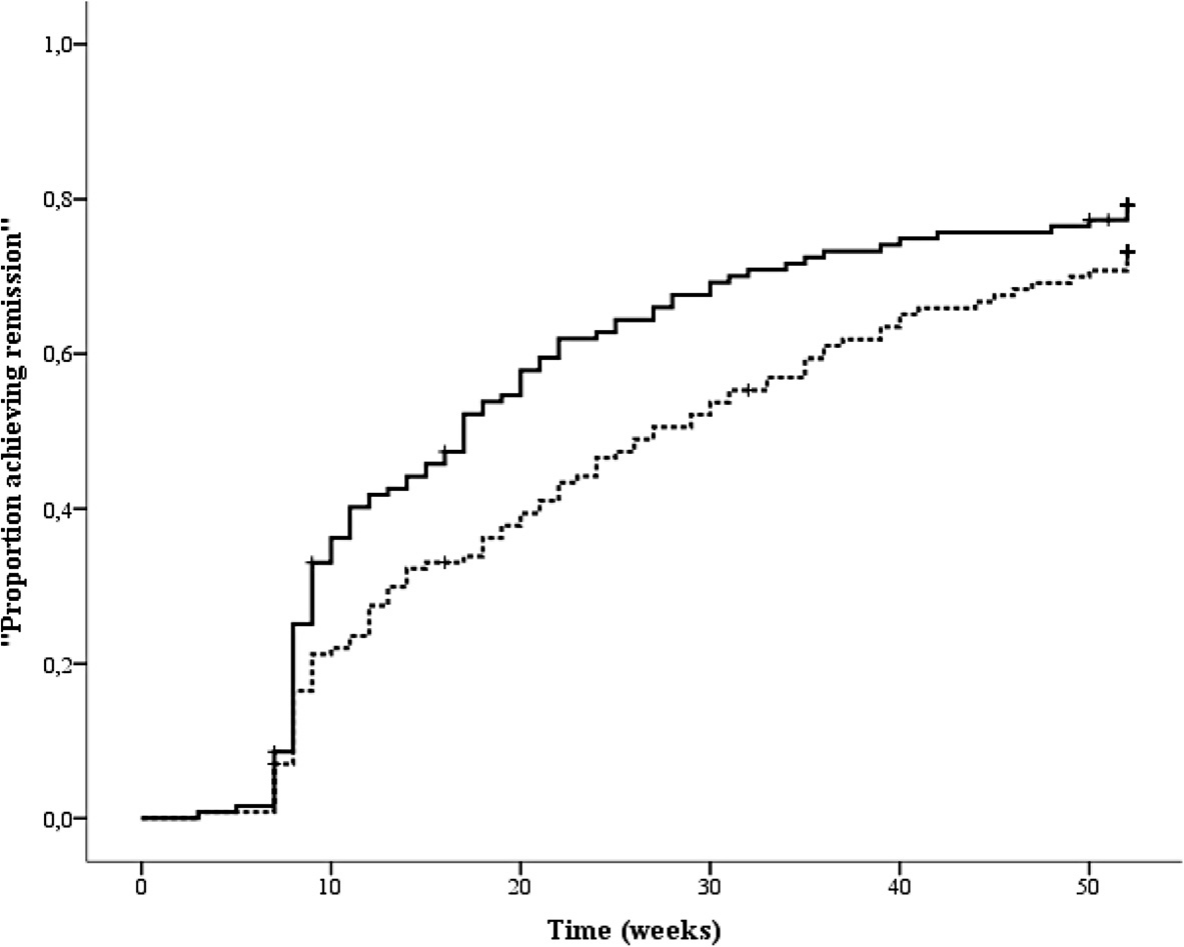
Kaplan-Meier curves for time until first remission was reached. The *solid line* represents cohort II, and the *dashed line* represents cohort I

First remission within 6 months was achieved in 63.3 % (81 of 128) of the patients in cohort II versus 48.4 % (62 of 128) in cohort I (*P* = 0.02). First remission within 12 months was achieved in 77.3 % (*n* = 99 of 128) of the patients in cohort II versus 71.9 % (92 of 128) in cohort I (*P* = 0.31). Median time until first remission was 17 weeks (95 % confidence interval [CI] 13.2–20.8) in cohort II versus 27 weeks (95 % CI 20.7–33.3) in cohort I (*P* = 0.04) (Fig. 1).

The proportion of patients in remission in cohorts II and I at the time of the 6-month assessments were 61.7 % (74 of 120) and 45.9 % (56 of 122) (*P* = 0.01), respectively. The proportion of patients in remission in cohorts II and I at the 12-month assessments were 63.6 % (77 of 121) and 60.3 % (*n* = 73 of 121) (*P* = 0.60). The majority of the patients in both cohorts reached their first remission using the initial medication only. In cohort II, 84.8 % achieved remission with MTX + HCQ, while in cohort I 63.0 % achieved remission with MTX monotherapy.

### Course over time of the DAS28

**Fig. 2:**
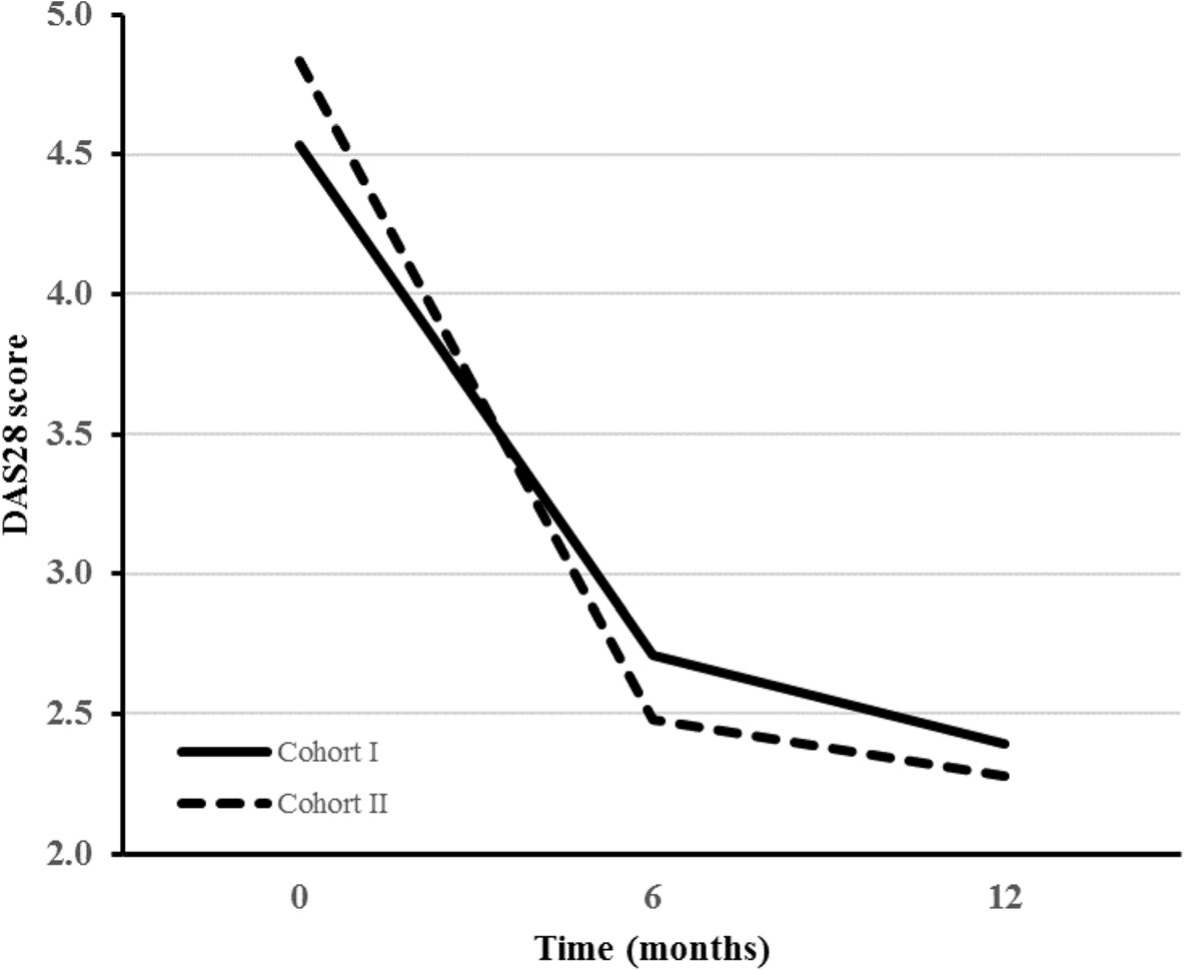
Decrease in mean 28-joint Disease Activity Score (DAS28) in treat to target cohort II versus cohort I in 1 year. The *dashed line* represents cohort II, and the *solid line* represents cohort I

Figure 2 represents the mean DAS28 over time in both cohorts. A significant time by strategy interaction was found (*P* = 0.01), indicating a different pattern of DAS28 change over time between both cohorts. At 6 months, the mean DAS28 in cohort II was below the cutoff for remission (DAS28 < 2.6), while the mean DAS28 in cohort I was still above remission (DAS28 > 2.6). At 12 months, mean DAS28 was <2.6 in both cohorts. According to the post hoc ANCOVA, mean DAS28 scores were significantly lower in cohort II at 6 months (mean difference 0.30, 95 % CI 0.01–0.58, *P* = 0.04) but not at 12 months (mean difference 0.13, 95 % CI −0.14 to 0.40, *P* = 0.36).

### Medication use

At baseline, 67 patients (53.3 %) in cohort II received an intramuscular injection with triamcinolone versus 4 patients (3.1 %) in cohort I (*P* < 0.01). Receiving an injection with triamcinolone in cohort II was associated with a higher odds ratio (OR 2.14, 95 % CI 1.03–4.46, *P* = 0.04) of achieving first remission within 6 months but no longer with achieving remission within 12 months (OR 1.77, 95 % CI 0.77–4.10, *P* = 0.018). The actual medication used at 12 months (Table 2) was significantly different between both cohorts (*P* = 0.03). The vast majority of the patients in both cohorts received conventional synthetic disease-modifying antirheumatic drugs (csDMARDs) only at 12 months. A small percentage of the patients in both cohorts were prescribed a biologic DMARD. In cohort II, there were slightly more patients for whom DMARD therapy was fully discontinued.

**Table 2:** Medication at 12 months

Medication	Cohort I (*n* = 128)	Cohort II (*n* = 128)
No DMARD	2 (1.6 %)	10 (7.8 %)
csDMARD only	116 (90.6 %)	104 (81.3 %)
bDMARD	5 (3.9 %)	11 (8.6 %)
Lost to follow-up	5 (3.9 %)	3 (2.3 %)

*DMARD* disease-modifying antirheumatic drug, *csDMARD* conventional synthetic disease-modifying antirheumatic drug, *bDMARD* biologic disease-modifying antirheumatic drug

## Discussion

The results of this study suggest that the previously reported outcomes that were achieved by implementation of T2T with initial MTX monotherapy in daily clinical practice may be reproduced, and even improved upon, using an initial combination approach. In the present study, DAS28 remission was reached at least once in 77.3 % of the patients in cohort II versus 71.9 % in cohort I in 1 year of follow-up. Although after 1 year there was no significant difference in the number of patients who had reached remission, remission was reached significantly more rapidly, with a median time to first remission of 17 weeks in cohort II versus 27 weeks in cohort I. A significant difference between strategies in mean DAS28 scores was also found at 6 months, but no longer at 12 months. Therefore, T2T using initial combination therapy may not be superior in the number of patients achieving remission in the longer term, but the strategy does lead to a significantly shorter time until first remission is achieved.

Researchers in various T2T studies in patients with recent-onset RA have reported remission rates varying from 10 % to 78 % [25]. However, it should be noted that these remission rates are difficult to compare, as the investigators in these studies used different criteria for remission and reported slightly different follow-up periods. The TICORA study had the highest proportion (65 %) of patients in remission (defined as DAS44 < 1.6) after 18 months of follow-up so far [4]. In the BeST study, the overall proportion of patients in DAS44 remission at 1 year was 32 %, with no differences between the four therapeutic groups [26]. The CAMERA group reported that 35 % of the patients in the intensive group attained remission (defined as no swollen joints and at least two of three of the following criteria: number of tender joints ≤3, ESR ≤20 mm/h, and VAS general well-being ≤20 mm) for a period of 3 months after 1 year of follow-up [3]. The IMPROVED study showed an early DAS44 remission rate of 61 % [15]. The CareRA trial researchers, comparing three different intensive combination strategies with glucocorticoids, reported DAS28 (based on CRP) remission rates ranging from 68.1 % to 73.6 % within 16 weeks [27]. The tREACH trial investigators compared two triple-DMARD induction therapies with MTX monotherapy and reported that approximately 78 % of the patients using triple-DMARD therapy had a DAS <2.4 after 3 months, compared with 60 % of the patients using MTX monotherapy [28].

The present study shows that both DREAM registry remission induction cohorts I and II resulted in comparably high proportions of patients achieving remission within 12 months. While the implementation of a protocol T2T may in itself explain the high remission rates in both cohorts, apparently even better results may be attained regarding the time needed to achieve first remission by implementing initial combination protocols in daily clinical practice. This is in accordance with previous randomized clinical trials. Among the different therapeutic strategies, early combination therapy that included high-dose step-down DMARDs and low-dose steroids was shown to be most effective in the management of early RA [8, 26, 29–31]. Low-dose prednisone added to conventional DMARD treatment in patients with early RA induced earlier and better disease activity control and provided greater probability for a more stable clinical remission over time [32]. A strict protocol, frequent monitoring, and the commitment and adherence of doctors and nurses may also contribute to optimal outcomes in daily clinical practice [6, 33].

Differences in disease activity and in time until remission are clinically important and are likely to also have significant social and economic impact. For instance, patients with persistently active disease are more likely to discontinue work [34]. The DREAM registry remission induction cohort I already showed the feasibility of sustained remission in daily clinical practice [16]. Cohort II also demonstrated impressive improvements in disease activity, comparable with or even better than that reported in previous studies of combination therapy for RA [5, 8, 11–13, 18]. In the present study, mean DAS28 scores were significantly lower in cohort II than in cohort I at 6 months but not at 12 months. This is in line with clinical trials showing that initial combination therapy appears to result in more rapid improvements in disease activity, daily functioning, and quality of life than initial monotherapy [4, 11, 26, 35].

Optimal use of MTX in combination with other DMARDs early in the course of the disease leads to considerable improvement in disease activity [3, 4, 19]. Unfortunately, previous studies show some conflicting results regarding the efficacy of monotherapy versus combination therapy. Double- or triple-combination therapy appears to lead to better clinical outcomes than MTX monotherapy [13, 26, 35]. The TEAR trial concluded that combination treatment was more effective than MTX monotherapy before step-up therapy [14]. The CARDERA study, which was not an initial combination therapy, showed that intensive combination therapy with MTX, ciclosporin, and glucocorticosteroids after failure of MTX monotherapy leads to sustained benefits in damage and disability outcomes [36]. However, a recent study done in Belgium (the CareRA trial) showed that in high-risk patients with RA, MTX in combination with a moderate step-down dose of glucocorticosteroids was as effective in inducing remission as DMARD combination therapies with moderate or high step-down glucocorticosteroid doses [27]. In contrast, the tREACH trial showed that a DMARD combination was better than MTX monotherapy, both in combination with low-dose glucocorticoid bridging [12]. In the United States, MTX + HCQ is by far the DMARD combination most commonly prescribed by rheumatologists [37]. It has been shown to be more potent than MTX used alone [38]. An explanation for this may be that HCQ increases the bioavailability of MTX and/or reduces the clearance of the drug [39].

In the present study, the vast majority of the patients in both cohorts achieved the treatment target using csDMARDs only, and a small number of the patients in both cohorts were using biologic DMARDs at 12 months. This highlights again the importance of the initial treatment choice for the majority of the patients.

It should be noted that the present study was a quasi-experimental study of two cohorts separated over time. The first cohort started in 2006 with MTX monotherapy, and the second cohort started in 2012 with a DMARD combination therapy. Although we did not conduct a randomized trial, we still think that the design and results of the study allow us to compare the two cohorts. The cohorts consist of very similar populations of all consecutive newly diagnosed patients with RA treated in the same hospitals by the same rheumatologists. Patients in cohort II were additionally case–control-matched with patients in cohort I on gender, age, and baseline disease activity. Although baseline disease activity, disability, and pain were slightly but significantly worse in cohort II, the number of patients achieving remission within 6 months was even higher in this cohort. Moreover, post hoc analyses of mean DAS28 scores controlling for baseline disease activity produced very similar results.

Because in our study we used real-life observational data, the results are generalizable to daily clinical practice. Longer follow-up is needed to investigate the sustainability of remission in cohort II. However, previous studies have shown that a shorter time until remission is related to the sustainability of remission, supporting the importance of early intervention with effective therapy to achieve early remission [40]. Although the remission rates in this study were very high, there is still a small percentage of patients who do not achieve remission, thus creating a need to further identify predictors of remission [21].

## Conclusions

Initial RA combination therapy with high-dose MTX, HCQ, and optional intramuscular TCA was not superior in achieving the number of patients in remission after 1 year, but it did result in a shorter time until first remission versus a step-up approach using MTX and SSZ in the setting of T2T therapy in daily clinical practice.

## Abbreviations

ANCOVA: analysis of covariance
Anti-CCP: anti- cyclic citrullinated peptide
bDMARD: biologic disease-modifying antirheumatic drug
BMI: body mass index
CI: confidence interval
CRP: C-reactive protein
csDMARD: conventional synthetic disease-modifying antirheumatic drug
DAS28: 28-joint Disease Activity Score
DMARD: disease-modifying antirheumatic drug
DREAM: Dutch Rheumatoid Arthritis Monitoring registry
ESR: erythrocyte sedimentation rate
HAQ-DI: Health Assessment Questionnaire Disability Index
HCQ: hydroxychloroquine
IQR: interquartile range
MCS: Mental Component Summary
MTX: methotrexate
OR: odds ratio
PCS: Physical Component Summary
RA: rheumatoid arthritis
RF: rheumatoid factor
SD: standard deviation
SF-36: 36-item Short Form Health Survey
SJC: swollen joint count
SSZ: sulfasalazine
T2T: treat to target
TCA: triamcinolone acetonide
TJC: tender joint count
TNF: tumor necrosis factor
VAS: visual analogue scale
